# Independent effect of prior exacerbation frequency and disease severity on the risk of future exacerbations of COPD: a retrospective cohort study

**DOI:** 10.1038/npjpcrm.2016.46

**Published:** 2016-09-08

**Authors:** Miguel Santibañez Margüello, Roberto Garrastazu, Mario Ruiz-Nuñez, Jose Manuel Helguera, Sandra Arenal, Cristina Bonnardeux, Carlos León, Marc Miravitlles, Juan Luis García-Rivero

**Affiliations:** 1 Preventive Medicine and Public Health Area, Universidad de Cantabria, IDIVAL, Santander, Spain; 2 Centro de Salud de Gama, Servicio Cántabro de Salud, Bárcena de Cicero, Spain; 3 Centro de Salud de Liérganes, Servicio Cántabro de Salud, Miera, Spain; 4 Centro de Salud Bajo Asón, Servicio Cántabro de Salud, Ampuero, Spain; 5 Centro de Salud de Suances, Servicio Cántabro de Salud, Suances, Spain; 6 Centro de Salud Campoo-Los Valles, Servicio Cántabro de Salud, Mataporquera, Spain; 7 Pneumology Department, Hospital Universitari Vall d’Hebron; CIBER of respiratory diseases (CIBERESP), Barcelona, Spain; 8 Pneumology Department, Hospital de Laredo, Servicio Cántabro de Salud, Laredo, Spain

## Abstract

Few studies have researched the independent effect of COPD severity on the risk of future exacerbations adjusted by previous exacerbation frequency. We aimed to analyse the independent effect of COPD severity on the risk of exacerbations in the following year, and whether this effect was stronger or not than the effect of a previous history of exacerbations. We conducted a retrospective population-based cohort study including 900 patients with confirmed COPD. Exacerbation frequency was observed for the previous year and for the following year. Patients were defined as ‘Frequent Exacerbator’ (FE) phenotype if they suffered ⩾2 exacerbations in a year, and were categorised according to the severity of COPD (GOLD Grades 1–4). Odds ratios (ORs) were estimated by logistic regression adjusting for age, gender, smoking status, severity of COPD and being FE in the previous year. The main predictor of being FE among all grades of COPD severity was a history of frequent exacerbations in the previous year: adjusted OR 4.97; 95% confidence interval (CI) (3.54–6.97). COPD severity was associated with a higher risk of being FE: Crude OR GOLD Grade 4 3.86; 95% CI (1.50–9.93). However, this association diminished after adjusting for being FE in the previous year: adjusted OR 2.08; 95% CI (0.75–5.82). Our results support that a history of frequent exacerbations in the previous year is the most important independent predictor of exacerbations in the following year, also among the most severe COPD patients. Severity of COPD would be associated with a higher risk of exacerbations, but this effect would be partly determined by the exacerbations suffered in the previous year.

## Introduction

Chronic obstructive pulmonary disease (COPD) is one of the most common lung conditions observed in clinical practice and the third leading cause of death in the world.^1^ The association of COPD and smoking is well established. However, an increasing number of studies have reported a significant prevalence of COPD among non-smokers.^2–4^

Exacerbations of COPD are episodes of worsening of symptoms that carry significant consequences for patients,^5–7^ being responsible for a large proportion of the health-care costs attributable to this prevalent condition.^8^ Consequently, their prevention is a key component of COPD-management strategies.^9,10^

Despite the importance of exacerbations, we know relatively little about their determinants, probably because the heterogeneity of COPD exacerbations reflects their dependence on a complex spectrum of multiple risk factors.^5^

The most consistent predictor of exacerbations appears to be a previous history of exacerbations.^11^ This would be supported by new large observational cohort studies such as ‘The Evaluation of COPD Longitudinally to Identify Predictive Surrogate Endpoints (ECLIPSE) Study’,^12^ potentially indicating a definable phenotype of exacerbation susceptibility. However, this observational study did not include milder forms of COPD (Mild-GOLD grade 1), nor did it include COPD-confirmed patients without a history of tobacco consumption.

Although exacerbations are generally considered to become more frequent as the severity of the underlying COPD increases,^13^ very few studies have researched the independent effect of COPD severity adjusted by a previous history of exacerbations.

We used the data from a retrospective population-based cohort study, including Mild-GOLD grade 1 or Non-smoker patients with confirmed COPD, to test whether a history of frequent exacerbations is the most important predictor of exacerbations in the following year, with independence of disease severity. In addition, we tried to analyse the independent effect of disease severity on the risk of susceptibility to exacerbations.

## Results

Among 900 patients with confirmed COPD, 194 (21.6%) were women. The overall mean age was 71.2 years (s.d. 11.0), with a mean of years since COPD diagnosis of 6.6 years (s.d. 5.5). In all, 15.7% of the COPD-confirmed patients were never-smokers. The baseline characteristics of the patients are reported in Table 1.

With regard to COPD severity, most of the patients were moderate-GOLD grade 2 (60%); 26.2% were severe-GOLD grade 3; 10.4% were mild-grade 1; and 3.5% were very severe-GOLD grade 4. Twenty eight per cent of the mild-GOLD grade 1 patients developed two or more exacerbations in the following year (‘FE’ phenotype). Incidence of ‘FE’ phenotype increased in our study with increasing disease severity (40%, 42% and 60% among GOLD grades 2, 3 and 4 were ‘FE’ phenotype, respectively).

In all, 85% of our sample was affected by at least one of the studied co-morbidities. The most prevalent co-morbidity was high blood pressure, affecting 60.2% of patients. See Supplementary Table 2.

Table 2 shows the associations between a history of exacerbations and the risk of suffering frequent exacerbations in the following year. Each exacerbation during the past 4 years (range 0–36) increased the risk of being ‘FE’ the next year by 1.20. Each exacerbation during the previous year (range 0–10) increased the risk of being ‘FE’ phenotype the next year by 1.76. As during 4 years the number of exacerbations was approximately four times as high as the number of exacerbations in a single year, this odds ratio (OR) of 1.20 would be equivalent to an average OR per year of 2.07 (2.07=1.20^4^).

A statistically significant dose–response p trend was obtained after ordinal-categorising the number of exacerbations during 2011 (adjusted linear *P* trend <0.001) into 0, 1 and⩾2 exacerbations. When categorising into ‘FE’ versus ‘Infrequent Exacerbations’ (IE) phenotype during the previous year, an adjusted Odds Ratio (ORa) of 4.97, 95% CI (3.54–6.97), was obtained with independence of COPD severity.

With respect to the stability of the frequent-exacerbation phenotype over time, among the 563 patients with infrequent exacerbations in the previous year, 438 also had infrequent exacerbations in the following year (negative predictive value, 77.8%). Among the 337 patients with frequent exacerbations in the previous year, there were 207 who had frequent exacerbations in the following year (positive predictive value, 61.4%); 85.5% of patients with frequent exacerbations in the previous year had at least one exacerbation in the following year. Thus, exacerbation frequency in the previous year had a sensitivity of 51.9% and a specificity of 85.8% for the frequency in the following year (see Table 3).

In addition, in Table 3 absolute risk reduction—also called risk difference—is presented. The risk difference was 38% (85.46 per cent—47.42 per cent=38 per cent). This means that if 100 patients were converted from FEs to infrequent exacerbators, 38% would be prevented from developing an exacerbation in the following year.

COPD severity according to forced expiratory volume in 1 s (FEV_1_) was associated with a higher risk of being ‘FE’ phenotype in the following year in our crude models: crude OR GOLD grade 4, 3.86; 95% CI (1.50–9.93) (see Table 4). After adjusting for age, gender and smoking status, results remained significant. However, when adding ‘Frequent of exacerbations in the previous year’ to the multivariable model, adjusted *p* trend did not yield statistical significance because GOLD grade 3 patients (ORa2 1.38) were not at a higher risk of being ‘FE’ phenotype than GOLD grade 2 patients (ORa2 1.41). Even the crude association for the very severe-GOLD grade 4 patients diminished and did not yield statistical significance: ORa2 2.08; 95% CI (0.75–5.82).

With respect to the remaining sociodemographic (age and gender), lifestyle (tobacco and BMI) and clinical variables (co-morbidities), only age was statistically significantly associated with a higher risk of being ‘FE’ phenotype in the previous year in the multivariable models (see Table 5). None of the studied co-morbidities was an independent predictor of ‘FE’ phenotype. See Supplementary Table 3.

## Discussion

### Main findings

The most important independent predictor of ‘Frequent Exacerbations’ in the following year was the exacerbation frequency during the previous year, with statistically significant dose–response patterns. This effect was independent of COPD severity and main confounders identified in our study. The number of exacerbations during the past 4 years (2008–2011) would also be a good independent predictor of ‘FE’ phenotype.

With regard to COPD severity, incidence of ‘FE’ phenotype’ increased in our study with increasing disease severity, and thus COPD severity was associated with ‘FE’ phenotype in the following year in our crude models. However, after adding ‘Frequency of exacerbations in the previous year’ as a confounding variable, the importance of disease severity as an independent predictor diminished. This strongly suggests that the number of exacerbations in the previous year is associated simultaneously with the COPD severity and the number of exacerbations in the following year. Thus, a history of exacerbations would be the most important predictor of ‘FE’ phenotype among all grades of disease severity, and even for the most advanced severity grades (very severe-GOLD grade 4) the exacerbations suffered in the past would act as an important intermediate step in the causal pathway of being ‘FE’ phenotype.

### Interpretation of findings in relation to previously published work

The importance of prior exacerbation frequency on the risk of future exacerbations is supported by the largest prospective cohort study (ECLIPSE Study)^12^ and numerous additional observational studies,^14–17^ as well as previous large intervention studies.^18–20^

With regard to COPD severity, some published studies support this rationale to some extent,^6,12,17,21^ but further studies with a higher sample size must focus on the real independent effect of COPD severity on the risk of exacerbation frequency.

In our sample, 10.4% of included patients were GOLD stage 1. In all, 28% of these mild-GOLD stage 1 patients developed two or more exacerbations in the following year (‘FE’ phenotype). These results provide clinical original information with respect to previous studies,^12,15^ supporting the hypothesis that there may therefore be a phenotype of exacerbation susceptibility that includes milder forms of COPD.

### Strengths and limitations of this study

One limitation is the retrospective study design based on secondary information available in clinical databases that were not specifically designed with this project in mind. In retrospective studies based on secondary information (records), a main limitation could be the low quality of that information; this could be either because of insufficient completion of medical records or lack of agreement among different records. To minimise any bias, we *a priori* decided to use only variables in which the data appeared systematically and objectively collected and in which the data appeared internally consistent. Where possible, we obtained agreement by making a comparison between the primary care and hospital records. In addition, it is important to indicate that all included records were checked for valid spirometric results confirming COPD diagnosis.

In some important variables such as FEV_1_, a non-negligible percentage of missing values was obtained (*N*=177, 19.7%) because the numeric specific value was not registered even though an obstructive spirometry was retrieved from the databases. We performed a sensitivity analysis concerning the effect of missing FEV_1_ data. We did so by treating missing values as a separate category and comparing the association between the missing values and the risk of suffering frequent exacerbations in the following year. The missing values showed greater similarity to the reference category (mild-GOLD grade 1) than to the more severe-GOLD grades 2–4. If those missing values were not actually unknown but were mild-GOLD grade 1, similar OR for the remaining GOLD grades 2–4 would have been obtained with a more precise 95% CI. This suggested that the missing data did not unduly bias our findings.

External validity is one of the main limitations regarding clinical trials.^22,23^ This problem can also affect observational studies based on strict inclusion and exclusion criteria.^24^ We checked that our final analysed sample was representative with respect to age, gender and primary health-care centre with respect to the base population. A major strength of our study is that our sample reflected a population-based (real-life) sample, and therefore mild stages and non-smokers with confirmed COPD were identified and included in the random sample. Our prevalence of 15.7% of confirmed COPD in never-smokers is also supported by published studies.^2–4^

The fact that information was based on records makes the existence of bias arising from the lack of blindness among patient's care providers (who treated patients retrospectively blinded to the development of the study) improbable. To minimise a selection bias, only COPD-confirmed cases were chosen, and an attempt to obtain the independent effect of predictors was made in the epidemiological and statistical approach by controlling confounding and stratified analysis.

### Implications for future research, policy and practice

Some patients with milder disease (GOLD stages 1) are also subject to frequent exacerbations, suggesting a distinct susceptibility phenotype. This highlights the necessity of reconsidering a more in-depth monitoring and therapeutic and preventive strategies in patients at GOLD grade 1 level classified as ‘FE’ phenotype.

### Conclusions

Our study supports the hypothesis that the most important independent predictor of ‘FE’ phenotype in the following year is a history of frequent exacerbations in the previous year, among all grades of COPD severity. Severity of COPD would be associated to a less extent with a higher risk of being ‘FE’ phenotype, and this effect would be in part determined by the exacerbations suffered in the previous year.

## Materials and methods

### Design and participants

This is a retrospective population-based cohort study. Methods have been described elsewhere.^25^ All the patients⩾35 years old, with prevalent codes ‘R91’ or ‘R95’ according to the lnternational Classification of Primary Care (ICP),^26^ were identified through electronic clinical databases in the province of Cantabria (Northern Spain) on 31 December 2011. The recruitment criteria included no restriction according to a history of tobacco consumption or degree of FEV_1_ impairment. A total of 9,334 potential COPD patients were identified out of the total population of 362,372 people registered in Cantabria.

**Figure 1:**
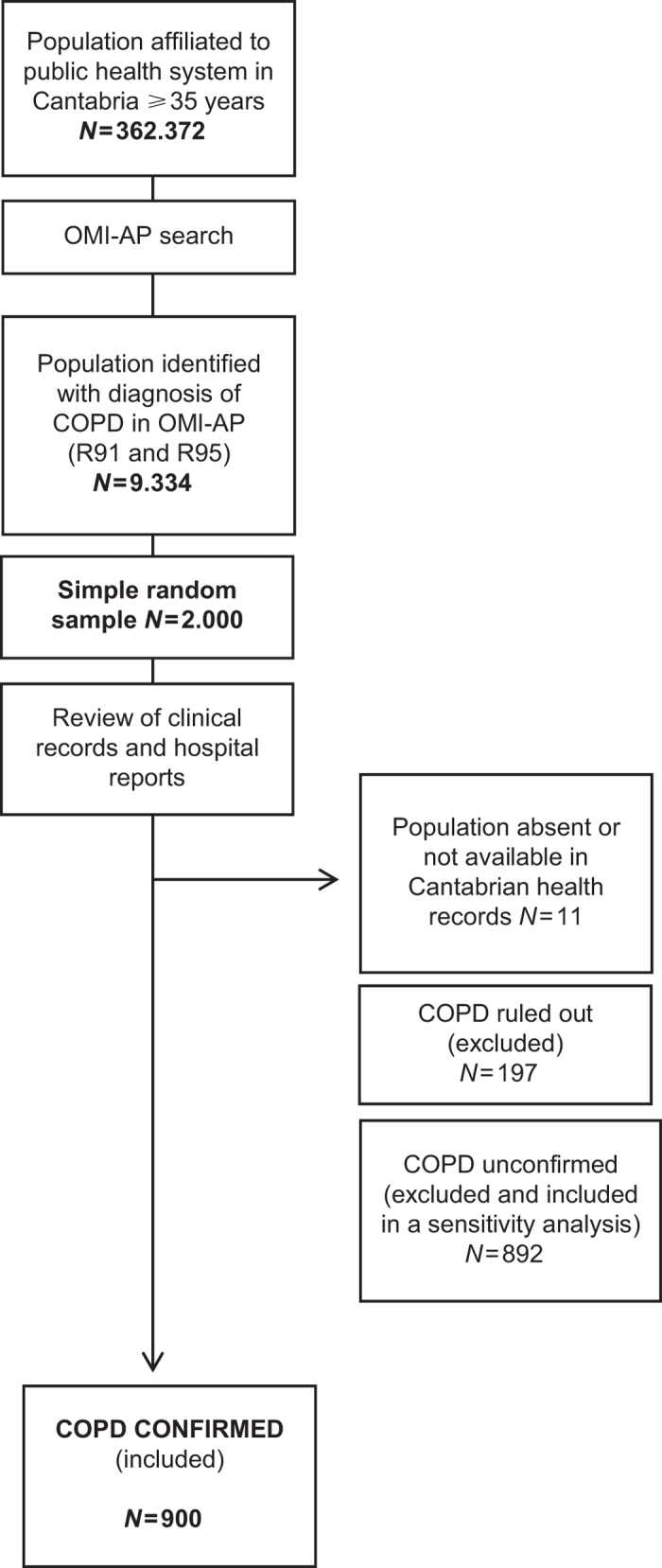
Flow chart for selection of the study sample.

A sample of 2,000 patients was obtained by simple random sampling. A comparison of the 2000 sampled patients with respect to the 9,334 potential COPD patients, on basic sociodemographics, is presented in Supplementary Table 1. Spirometric data were carefully revised for each of these patients. COPD diagnosis was considered as confirmed if a spirometry with bronchodilatation test consistent with obstructive disease (defined as a FEV_1_/forced vital capacity ratio of <0.7) was retrieved from the databases, and it was rejected if spirometry was not consistent with obstructive disease. Diagnosis of COPD was confirmed in 900 patients (45.3%), rejected in 197 patients (9.9%) and not confirmed—not rejected in the remaining patients (44.8%). Final analysis was restricted to COPD-confirmed patients. The flow chart for selecting study patients is shown in Figure 1.

### Data sources and variables

Data for each patient were individually gathered from primary care medical electronic medical records (OMI-AP)^27–29^ and complemented with hospital electronic records (eVISOR), which contain information about urgent assistance, consultations done by lung specialists and hospital admissions.

Clinical and demographic characteristics of all patients were recorded, including age, years since diagnosis of COPD, treatments and vaccinations, smoking status, alcohol consumption and co-morbidities.

Records of diagnoses of selected co-morbidities,^30,31^ including diabetes, osteoporosis, atrial fibrillation, ischaemic heart disease, heart failure and high blood pressure, occurring before the start of the study, were retrieved from the database.

Exacerbations were defined as events that led a care provider to prescribe antibiotics or corticosteroids (or both; moderate exacerbations) or that led to hospitalisation (severe exacerbations). This case definition therefore met the criteria for a definition of health-care utilisation.^12,32,33^ Exacerbation frequency was observed for the previous year (2011), the previous four years (2008–2011) and for the following year (2012). We also defined frequent exacerbations (‘FE’ phenotype) as two or more exacerbations in a year, because this definition coincides with current health-care utilisation criteria for frequent exacerbations.^12,32^

### Statistical analyses

Discrete variables were expressed as counts (percentage) and continuous variables as mean (s.d.). Statistical differences between groups were assessed with the *χ*^2^-test or Fisher’s exact test, when appropriate, for categorical variables. The Student’s *t*-test was used for the continuous variables.

Exacerbation frequency (both moderate and severe exacerbations) for the following year (2012) was treated as a dichotomous dependent variable in the regression models: ‘FE’ phenotype versus ‘Infrequent Exacerbator’ (IE) phenotype (none or one exacerbation).

Exacerbation frequencies ‘during the previous year (2011)’ and ‘during the last four years (2008–2011)’ were treated as independent variables and included as continuous variables in the regression models.

Exacerbation frequency ‘during the previous year (2011)’ was also ordinal-categorised as ‘0 exacerbations’, ‘1 exacerbation’ and ‘⩾2 exacerbations’. Next, the ‘0’ and ‘1’ categories were combined, and thus a dichotomous variable ‘FE’ versus ‘IE’ phenotype in the previous year was obtained.

Patients were also ordinal-categorised into 4 categories of severity of COPD according to FEV_1_ quantitative results, as defined by the Global Initiative for Chronic Obstructive Lung Disease (GOLD grades 1–4).^34^ Last, FEV_1_ was transformed in an ordinal scale according to per 5% decrease in per cent of predicted value.^12^

Crude and adjusted ORs with their 95% CI were estimated by unconditional logistic regression, adjusting for age (continuous), gender, smoking status (no smoker, former smoker, current smoker), severity of COPD (ordinal GOLD grades 1–4) and ‘FE’ phenotype (yes/no) in the previous year.

Tests for OR trends were calculated for the ordinal independent variables using logistic models that included categorical terms as continuous variables. For these trend tests, we used the likelihood ratio test.

The final sample size (*n*=900) would have sufficient power (1−*β*>90%) to detect relative risks ⩾1.16 as significant for a 50% risk of the effect in the unexposed, considering an unexposed/exposed ratio of 1, using a two-tailed *χ*^2^-test with an *α* level <0.05.

To assess the stability of the frequent-exacerbation phenotype over time, we calculate positive and negative predictive values, sensitivity and specificity. As impact measure, the absolute risk reduction, also called risk difference, was estimated.

The *α* error was set at 0.05, and all *P* values were bilateral. All statistical analyses were conducted using IBM SPSS Statistics version 22.0.

Approval of the research protocol was obtained from the Clinical Research Ethics Committee of Cantabria before the acquisition of data. Patient records/information was anonymised and de-identified before analysis.

## Figures and Tables

**Table 1:** Baseline sociodemographic, lifestyle and clinical characteristics of the patients

	*IE*		*FE*		*Total*		P *value*
	N*=568*	*Row %*	N*=332*	*Row %*	N*=900*	*Column %*	
Age, mean [s.d.]	69.8	[11.4]	73.6	[9.8]	71.2	[11.0]	<0.001
							
*Gender*							
Female	123	63.4	71	36.6	194	21.6	0.924
Male	445	63.0	261	37.0	706	78.4	
							
Year since COPD diagnosis, mean [s.d.]	6.2	[5.1]	7.4	[6.1]	6.6	[5.5]	0.001
							
*Smoking*							
Never-smoker	84	62.2	51	37.8	135	15.7	0.001
Former smokers	289	57.9	210	42.1	499	57.9	
Current smokers	166	72.8	62	27.2	228	26.5	
Missing values	29	76.3	9	23.7	38	4.2	
							
*BMI, mean [s.d.]*	28.8	[4.7]	28.9	[5.3]	28.9	[4.9]	0.89
Normal weight (18.5–24.9)	91	59.9	61	40.1	152	18.8	0.396
Overweight (25–29.9)	198	63.9	112	36.1	310	38.4	
Obesity (⩾30)	213	63.4	123	36.6	336	41.6	
Underweight (<18.5)	4	40	6	60	10	1.2	
Missing values	62	67.4	30	32.6	92	10.2	
							
*BODE-BMI*^a^							
BMI >21	491	62.9	290	37.1	781	96.7	0.44
BMI ⩽21	15	55.6	12	44.4	27	3.3	
Missing values	62	67.4	30	32.6	92	10.2	
							
*GOLD Grades (FEV_1_)*							
Grade 1 (⩾80%)	54	72	21	28	75	10.4	0.029
Grade 2 (⩾50–80%)	259	59.8	174	40.2	433	59.9	
Grade 3 (⩾30–49.9%)	110	57.9	80	42.1	190	26.3	
Grade 4 (<30%)	10	40	15	60	25	3.5	
Missing values	135		42		177		
							
*History of exacerbations*							
Number of exacerbations in the previous year, mean [s.d.]	0.92	[1.20]	2.39	[2.01]	1.46	[1.71]	<0.001
Number of exacerbations in the previous 4 years, mean [s.d.]	3.1	[3.56]	7.49	[6.32]	4.72	[5.21]	<0.001
							
*Number of exacerbations in the previous year*							
None (0 exacerbations)	275	84.6	50	15.4	325	36.1	<0.001
1 exacerbation	163	68.3	75	31.7	238	26.5	
⩾2 exacerbations	130	38.6	207	61.4	337	37.4	
							
*History of severe exacerbations (hospitalised for exacerbation) in the previous year*							
Number of COPD admissions, mean [s.d.]	0.17	[0.51]	0.51	[1.03]	0.29	[0.76]	<0.001
None (0 COPD admissions)	497	68.1	233	31.9	730	81.1	<0.001
1 COPD admission	54	47	61	53	115	12.8	
⩾2 COPD admissions	17	30.9	38	69.1	55	6.1	

Abbreviations: BMI, body mass index; COPD, chronic obstructive pulmonary disease; FE, frequent exacerbations in the following year; FEV_1_, forced expiratory volume in 1 s; IE, infrequent exacerbations in the following year.

a Risk cutoff point for BMI according to BODE index.

**Table 2:** Crude and adjusted ORs according to previous exacerbations for the risk of suffering frequent exacerbations in the following year

	*IE*	*FE*	*ORc*	*95% CI*	*ORa*	*95% CI*
	N*=568*	N*=332*				
*History of exacerbations*						
Number of exacerbations in the previous year (continuous)	—	—	1.85	1.66–2.06	1.76	1.56–2.00
Number of exacerbations in the previous four years (continuous)	—	—	1.24	1.19–1.30	1.20	1.15–1.26
						
*Frequent exacerbations in the previous year*						
Infrequent exacerbations (⩽1)	438	125	1	—	1	—
Frequent exacerbations (⩾2)	130	207	5.58	4.15–7.50	4.97	3.54–6.97
						
*Number of exacerbations in the previous year*						
None (0 exacerbations)	275	50	1	—	1	—
1 exacerbation	163	75	2.53	1.69–3.80	1.86	1.18–2.95
⩾2 exacerbations	130	207	8.76	6.03–12.71	6.77	4.45–10.28
Linear *P* trend			<0.001		<0.001	
						
*History of severe exacerbations (hospitalised for exacerbation) in the previous year*						
Number of COPD admissions (continuous)	—	—	1.91	1.53–2.38	1.13	0.93–1.37
						
*Frequent severe exacerbations in the previous year*						
None (0 COPD admissions)	497	233	1	—	1	—
⩾1 COPD admissions	71	99	2.97	2.11–4.19	2.13	1.45–3.14
						
*Number of severe exacerbations in the previous year*						
None (0 COPD admissions)	497	233	1	—	1	—
1 COPD admission	54	61	2.41	1.62–3.59	1.75	1.12–2.72
⩾2 COPD admissions	17	38	4.77	2.64–8.63	3.35	1.75–6.41
Linear *P* trend			<0.001		<0.001	

Abbreviation: CI, confidence interval; COPD, chronic obstructive pulmonary disease; FE, frequent exacerbations in the following year; IE, infrequent exacerbations in the following year; OR, odds ratio; ORc, crude OR; ORa, adjusted OR by age, gender, smoking status and COPD severity (GOLD grades 1–4).

**Table 3:** Stability of the frequent-exacerbation phenotype over time

	*Number of exacerbations in the following year*									
	*0*	*⩾1*	*Total*	*Risk (%)*	*RD^b^*	*95% CI*	*Se (%)*	*Sp (%)*	*PV+ (%)*	*PV− (%)*
	N*=345*	N*=555*	N*=900*							
*Frequent exacerbations in the previous year*										
Infrequent exacerbations ⩽1)	296	267	563	47.42						
Frequent exacerbations (⩾2)	49	288	337	85.46	38.04	32.45–43.62	51.89	85.8	85.46	52.58
										
	*⩽1 (IE)*	*⩾2 (FE)*	*Total*	*Risk (%)*	*RD*	*95% CI*	*Se (%)*	*Sp (%)*	*PV+ (%)*	*PV- (%)*
	N*=568*	N*=332*	N*=900*							
*Frequent exacerbations in the previous year*										
Infrequent exacerbations (⩽1)	438	125	563	22.2						
Frequent exacerbations (⩾2)	130	207	337	61.42	39.22	32.99–45.45	62.35	77.11	61.42	77.8

Abbreviations: CI, confidence interval; FE, frequent exacerbations in the following year; IE, infrequent exacerbations in the following year; PV(+), positive predictive values; PV(−), negative predictive values; RD, risk difference with its 95% CI; Se, sensitivity; Sp, specificity.

**Table 4:** Crude and adjusted odds ratios according to COPD severity for the risk of suffering frequent exacerbations in the following year

*COPD severity according to FEV_1_*	*IE*	*FE*	*ORc*	*95% CI*	*ORa1*	*95% CI*	*ORa2*	*95% CI*
	N*=568*	N*=332*						
FEV_1_—per 5% decrease in % of predicted value	—	—	1.08	1.03–1.13	1.12	1.07–1.16	1.04	0.99–1.10
FEV_1_—mild-GOLD grade 1 (reference category)	54	21	1	—	1	—	1	—
FEV_1_—moderate-GOLD grade 2	259	174	1.73	1.01–2.96	1.64	0.93–2.88	1.41	0.77–2.56
FEV_1_—severe-GOLD grade 3	110	80	1.87	1.05–3.34	1.84	1.00–3.38	1.38	0.72–2.64
FEV_1_—very severe-GOLD grade 4	10	15	3.86	1.50–9.93	3.60	1.37–9.44	2.08	0.75–5.82
Linear *P* trend			0.01		<0.001		0.286	
Missing values	135	42	0.80	0.43–1.47	0.82	0.43–1.57	0.82	0.40–1.67

Abbreviations: CI, confidence interval; FE, frequent exacerbations in the following year; FEV_1_, forced expiratory volume in 1 s; IE, infrequent exacerbations in the following year; OR, odds ratio; ORc, crude OR; ORa1, adjusted OR by age, gender and smoking status; ORa2, adjusted OR adding to the multivariable model: FE phenotype (yes/no) in the previous year.

**Table 5:** Crude and adjusted ORs according to sociodemographic characteristics, for the risk of suffering frequent exacerbations in the following year

	*IE*	*FE*	*ORc*	*95% CI*	*ORa*	*95% CI*
	N*=568*	N*=332*				
Age**—**per 10-year increase	—	—	1.35	1.19–1.53	1.27	1.07–1.5
						
*Gender*						
Female	123	71	1	—	1	—
Male	445	261	1.02	0.73–1.41	1.07	0.68–1.68
						
*Smoking*						
Never-smoker	84	51	1	—	1	—
Former smokers	289	210	1.2	0.81–1.77	1.11	0.66–1.89
Current smokers	166	62	0.62	0.39–0.97	0.76	0.42–1.39
Linear *P* trend			0.009		0.228	
						
*BMI*						
Normal weight (18.5–24.9)	91	61	1	—	1	—
Overweight (25–29.9)	198	112	0.84	0.57–1.26	0.85	0.51–1.41
Obesity (⩾30)	213	123	0.86	0.58–1.28	0.82	0.5–1.34
Underweight (<18.5)	4	6	2.24	0.61–8.26	0.83	0.19–3.58
Linear *P* trend			0.831		0.463	
						
*BODE-BMI*^a^						
BMI >21	484	285	1	—	1	—
BMI ⩽21	22	17	1.31	0.68–2.51	1.47	0.66–3.26

Abbreviations: BMI, body mass index; CI, confidence interval; COPD, chronic obsrtuctive pilmonary disease; FE, frequent exacerbations in the following year; IE, infrequent exacerbations in the following year; OR, odds ratio; ORa, adjusted OR by age, gender, smoking status, COPD severity (GOLD grades 1–4) and FE phenotype (yes/no) in the previous year; ORc, crude OR.

a Risk cutoff point for BMI according to BODE index.
