# Supplementary material for: Independent effect of prior exacerbation frequency and disease severity on the risk of future exacerbations of COPD: a retrospective cohort study
Source: NPJ Prim Care Respir Med. 2016 Sep 8;26:16046–. doi: 10.1038/npjpcrm.2016.46 (PMC5015428; doi:10.1038/npjpcrm.2016.46)
Supplement: Supplementary Tables [file npjpcrm201646-s1.doc]

Suppl T1. Comparison of the 2000 sampled patients with respect to the 9,334 potential COPD patients, on basic socio-demographics

|  | **Potential COPD patients (N=9,334)** | **Simple random sample (N=2,000)** |
| --- | --- | --- |
| **Age**: Mean [SD] | 71.3 [12.3] | 70.9 [12.2] |
| **Years since diagnosis**: Mean [SD] | 6.5 [6.1] | 6.6 [6.6] |
| **Men**: N (%) | 7.188 (73.5%) | 1.473 (73.7%) |
| **Women**: N (%) | 2.596 (26.5%) | 5.27 (26.3%) |
| **Relation Men/Women** | 3:1 | 3:1 |

Suppl T2. Baseline clinical characteristics (co-morbidities) of the patients.

|  | **IE**^a^ | | **FE**^a^ |  | **Total** |  |  |
| --- | --- | --- | --- | --- | --- | --- | --- |
|  | **N=568** | **row %** | **N=332** | **row %** | **N=900** | **Column %** | ***p value*** |
| **co-morbidities** |  |  |  |  |  |  |  |
| None | 101 | 74.8 | 34 | 25.2 | 135 | 15 | *0.002* |
| At least one of the co-morbidities below | 467 | 61 | 298 | 39 | 765 | 85 |  |
| **Atrial Fibrillation** |  |  |  |  |  |  |  |
| No | 477 | 65.8 | 248 | 34.2 | 725 | 80.6 | *0.001* |
| Yes | 91 | 52 | 84 | 48 | 175 | 19.4 |  |
| **Ischemic heart disease** |  |  |  |  |  |  |  |
| No | 486 | 65.4 | 257 | 34.6 | 743 | 82.6 | *0.002* |
| Yes | 82 | 52.2 | 75 | 47.8 | 157 | 17.4 |  |
| **Heart Failure** |  |  |  |  |  |  |  |
| No | 480 | 66.5 | 242 | 33.5 | 722 | 80.2 | *<0.001* |
| Yes | 88 | 49.4 | 90 | 50.6 | 178 | 19.8 |  |
| **Any severe heart disease ^b^** |  |  |  |  |  |  |  |
| No | 377 | 67.8 | 179 | 32.2 | 556 | 61.8 | *<0.001* |
| Yes | 191 | 55.5 | 153 | 44.5 | 344 | 38.2 |  |
| **High Blood Pressure** |  |  |  |  |  |  |  |
| No | 241 | 67.3 | 117 | 32.7 | 358 | 39.8 |  |
| Yes | 327 | 60.3 | 215 | 39.7 | 542 | 60.2 | *0.02* |
| **Diabetes** |  |  |  |  |  |  |  |
| No | 428 | 65.6 | 224 | 34.4 | 652 | 72.4 | *0.007* |
| Yes | 140 | 56.5 | 108 | 43.5 | 248 | 27.6 |  |
| **Osteoporosis** |  |  |  |  |  |  |  |
| No | 529 | 64.8 | 287 | 35.2 | 816 | 90.7 | *0.001* |
| Yes | 39 | 46.4 | 45 | 53.6 | 84 | 9.3 |  |
| **Psiquiatric History** |  |  |  |  |  |  |  |
| No | 382 | 64.4 | 211 | 35.6 | 593 | 65.9 | *0.145* |
| Yes | 186 | 60.6 | 121 | 39.4 | 307 | 34.1 |  |
| **Lung Neoplasm** |  |  |  |  |  |  |  |
| No | 547 | 63.7 | 312 | 36.3 | 859 | 95.4 | *0.075* |
| Yes | 21 | 51.2 | 20 | 48.8 | 41 | 4.6 |  |
| **Metabolic Syndrome ^e^** |  |  |  |  |  |  | *0.386* |
| No | 536 | 63.3 | 311 | 36.7 | 847 | 94.1 |  |
| Yes | 32 | 60.4 | 21 | 39.6 | 53 | 5.9 |  |

^a^ IE denotes 'Infrequent Exacerbations (≤1)' the following year. FE denotes 'Frequent Exacerbations (≥2)' the following year.

^b^ Any severe heart disease : Ischemic heart disease. Heart Failure. Atrial Fibrillation.

^c^ Metabolic Syndrome: BMI ≥30, Diabetes mellitus, High Blood Pressure, use of statins as ‘subrogate’ of dyslipidemia.

Suppl T3. Crude and adjusted odds ratios according to co-morbidities, for the risk of suffering frequent exacerbations in the following year.

|  | **IE^a^** | **FE** |  | |  |  |  |  |
| --- | --- | --- | --- | --- | --- | --- | --- | --- |
|  | **N=568** | **N=332** | **ORc^b^** | **(95%** | **CI)** | **ORa^c^** | **(95%** | **CI)** |
| **co-morbidities** |  |  |  |  |  |  |  |  |
| None | 101 | 34 | 1 | -- |  | 1 | -- |  |
| At least one of the co-morbidities below | 467 | 298 | 1.9 | 1.25 | 2.87 | 1.31 | 0.77 | 2.22 |
| **Atrial Fibrillation** |  |  |  |  |  |  |  |  |
| No | 477 | 248 | 1 | -- |  | 1 | -- |  |
| Yes | 91 | 84 | 1.78 | 1.27 | 2.48 | 1.26 | 0.83 | 1.92 |
| **Ischemic heart disease** |  |  |  |  |  |  |  |  |
| No | 486 | 257 | 1 | -- |  | 1 | -- |  |
| Yes | 82 | 75 | 1.73 | 1.22 | 2.45 | 1.31 | 0.85 | 2.02 |
| **Heart Failure** |  |  |  |  |  |  |  |  |
| No | 480 | 242 | 1 | -- |  | 1 | -- |  |
| Yes | 88 | 90 | 2.03 | 1.46 | 2.83 | 1.47 | 0.97 | 2.21 |
| **Any severe heart disease ^d^** |  |  |  |  |  |  |  |  |
| No | 377 | 179 | 1 | -- |  | 1 | -- |  |
| Yes | 191 | 153 | 1.69 | 1.28 | 2.23 | 1.24 | 0.87 | 1.76 |
| **High Blood Pressure** |  |  |  |  |  |  |  |  |
| No | 241 | 117 | 1 | -- |  | 1 | -- |  |
| Yes | 327 | 215 | 1.35 | 1.02 | 1.79 | 1.02 | 0.71 | 1.45 |
| **Diabetes** |  |  |  |  |  |  |  |  |
| No | 428 | 224 | 1 | -- |  | 1 | -- |  |
| Yes | 140 | 108 | 1.47 | 1.09 | 1.99 | 1.24 | 0.86 | 1.79 |
| **Osteoporosis** |  |  |  |  |  |  |  |  |
| No | 529 | 287 | 1 | -- |  | 1 | -- |  |
| Yes | 39 | 45 | 2.13 | 1.35 | 3.34 | 1.76 | 0.98 | 3.16 |
| **Psiquiatric History** |  |  |  |  |  |  |  |  |
| No | 382 | 211 | 1 | -- |  | 1 | -- |  |
| Yes | 186 | 121 | 1.18 | 0.89 | 1.56 | 1.14 | 0.79 | 1.64 |
| **Lung Cancer** |  |  |  |  |  |  |  |  |
| No | 547 | 312 | 1 | -- |  | 1 | -- |  |
| Yes | 21 | 20 | 1.67 | 0.89 | 3.13 | 1.64 | 0.79 | 3.42 |
| **Metabolic Syndrome ^e^** |  |  |  |  |  |  |  |  |
| No | 536 | 311 | 1 | -- |  | 1 | -- |  |
| Yes | 32 | 21 | 1.13 | 0.64 | 2 | 1.16 | 0.59 | 2.25 |

^a^ IE denotes 'Infrequent Exacerbations (≤1)' the following year. FE denotes 'Frequent Exacerbations (≥2)' the following year.

^b^ Odds Ratio and 95% Confidence Intervals. ORc denotes “crude Odds ratio”

^c^ ORa denotes “adjusted OR” by Age, Gender, Smoking status, COPD severity (GOLD Grades 1-4) and frequent exacerbator phenotype (yes/no) the previous year.

d Any severe heart disease : Ischemic heart disease. Heart Failure. Atrial Fibrillation.

e Metabolic Syndrome: BMI ≥30, Diabetes mellitus, High Blood Pressure, use of statins as ‘subrogate’ of dyslipidemia.
